# NLRP3 priming due to skin damage precedes LTP allergic sensitization in a mouse model

**DOI:** 10.1038/s41598-022-07421-y

**Published:** 2022-02-28

**Authors:** Diego Pazos-Castro, Zulema Gonzalez-Klein, Alma Yuste Montalvo, Guadalupe Hernandez-Ramirez, Alejandro Romero-Sahagun, Vanesa Esteban, Maria Garrido-Arandia, Jaime Tome-Amat, Araceli Diaz-Perales

**Affiliations:** 1 Centro de Biotecnología y Genómica de Plantas (CBGP), Instituto Nacional de Investigación y Tecnología Agraria y Alimentaria (INIA), Universidad Politécnica de Madrid (UPM), Madrid, Spain; 2 Departamento de Biotecnología-Biología Vegetal, Escuela Técnica Superior de Ingeniería Agronómica, Alimentaria y de Biosistemas, Universidad Politécnica de Madrid (UPM), Madrid, Spain; 3 IIS-Fundación Jiménez Díaz, Universidad Autónoma de Madrid, Madrid, Spain

**Keywords:** Immunology, Molecular biology

## Abstract

Allergic sensitization is initiated by protein and epithelia interaction, although the molecular mechanisms leading this encounter toward an allergic phenotype remain unknown. Here, we apply the two-hit hypothesis of inflammatory diseases to the study of food allergy sensitization. First, we studied the effects of long-term depilation in mice by analyzing samples at different time points. Several weeks of depilation were needed until clear immunological changes were evidenced, starting with upregulation of NLRP3 protein levels, which was followed by overexpression of *Il1b* and *Il18* transcripts. Secondly, we assessed the effects of allergen addition (in this case, Pru p 3 in complex with its natural lipid ligand) over depilated skin. Systemic sensitization was evaluated by intraperitoneal provocation with Pru p 3 and measure of body temperature. Anaphylaxis was achieved, but only in mice sensitized with Prup3_complex and not treated with the NLRP3 inhibitor MCC950, thus demonstrating the importance of both hits (depilation + allergen addition) in the consecution of the allergic phenotype. In addition, allergen encounter (but not depilation) promoted skin remodeling, as well as CD45+ infiltration not only in the sensitized area (the skin), but across several mucosal tissues (skin, lungs, and gut), furtherly validating the systemization of the response. Finally, a low-scale study with human ILC2s is reported, where we demonstrate that Prup3_complex can induce their phenotype switch (↑CD86, ↑S1P1) when cultured in vitro, although more data is needed to understand the implications of these changes in food allergy development.

## Introduction

Food allergy is a chronic inflammatory condition affecting 8% of children and 5% of adults with no definitive treatment available^1^. Management of the disease consists of allergen restriction, with many complications due to allergen cross-reactivity and food processing cross-contamination that can lead to life-threatening symptoms, such as anaphylaxis^2,3^. Thus, deeper knowledge about the sensitization mechanism is still needed to design better treatments that could improve the quality of life of patients^4^.

The two-hit hypothesis was initially assembled to explain inflammatory mechanisms underlying some chronic pathologies^5,6^. Referring to allergies, we propose that a first hit could induce a maintained low inflammatory response, while the second one might initiate the allergic sensitization, which is a specific (against a particular allergen) and systemic (present in the whole organism) response. However, detailed molecular contribution of both events is still lacking^7^.

As previously reported, cutaneous exposure to food allergens in children and mice predisposes to IgE-mediated anaphylaxis^8,9^. Within this context, adjuvant-free murine models of cutaneous food allergy sensitization have been successfully developed, providing powerful tools to study the physiopathology of the disease^10,11^. Besides, longitudinal studies with human patients have shown that loss-of-function mutations in the filaggrin gene (*Flg*), which plays a crucial role in maintaining skin integrity, have been associated with a higher prevalence of food allergy^12^. Accordingly, depilation (as a model of skin abrasion) might play an important role in allergic sensitization, making the skin more susceptible to food allergy development.

On the other hand, lipids have been described as adjuvants in allergy^13–15^ and other type 2 (T2) pathologies^16^. In many cases, they have been reported as modulators of the interaction between allergens and mucosa^17,18^. In fact, some lipid transfer proteins (LTPs) show immunomodulator effects in humans, such as wheat’s TdLTP4, which reduces the inflammatory capacity of human macrophages^19^.

Over the past decades, it has been shown that allergen encounter induces the secretion of alarmins (IL25, IL33) by epithelial cells and keratinocytes, promoting pro-allergic responses mediated by dendritic cells (DCs), mast cells, eosinophils, and type 2 innate lymphoid cells (ILC2s), among others^20,21^. ILC2s are strong responders to alarmin signaling^22^, acting as central players in atopic diseases and anti-helminth reactions, as well as spreading T2 responses between distant mucosal sites^23,24^. Also, sphingosine-1-phosphate (S1P) has been recently described as an important regulator of these ILC2s, determining their migration patterns following a S1P gradient through the organism^25^.

Although in vivo models have broadened our knowledge of skin-mediated food allergy sensitization, we still ignore the molecular mechanisms underlying this event, as well as the contribution of lipid ligands in the process. Within this context, we produced a mouse model of sensitization using the major peach allergen, the LTP Pru p 3^26^, which is physiologically found as a protein-lipid complex (from now on, “Prup3_complex”). This lipid consists of an alkaloid fraction bound to phytosphingosine^27,28^. Our group has demonstrated that the presence of this sphingosine-like lipid ligand is essential to induce an immunological response in humans and mice^10,29^. Here, we show that depilation causes the priming of the inflammasome pathway, while the presence of the allergen (Pru p 3), specially with its ligand, favors the systemic anaphylactic phenotype. Also, our observations suggest a crosstalk between the skin and other mucosal sites. Our results are directly relevant to food allergy in patients with dermal pathologies, especially in the early ages.

## Results

### First hit: effects of the depilation

#### Depilation induces epithelial permeability and NLRP3 priming in murine skins

**Figure 1:**
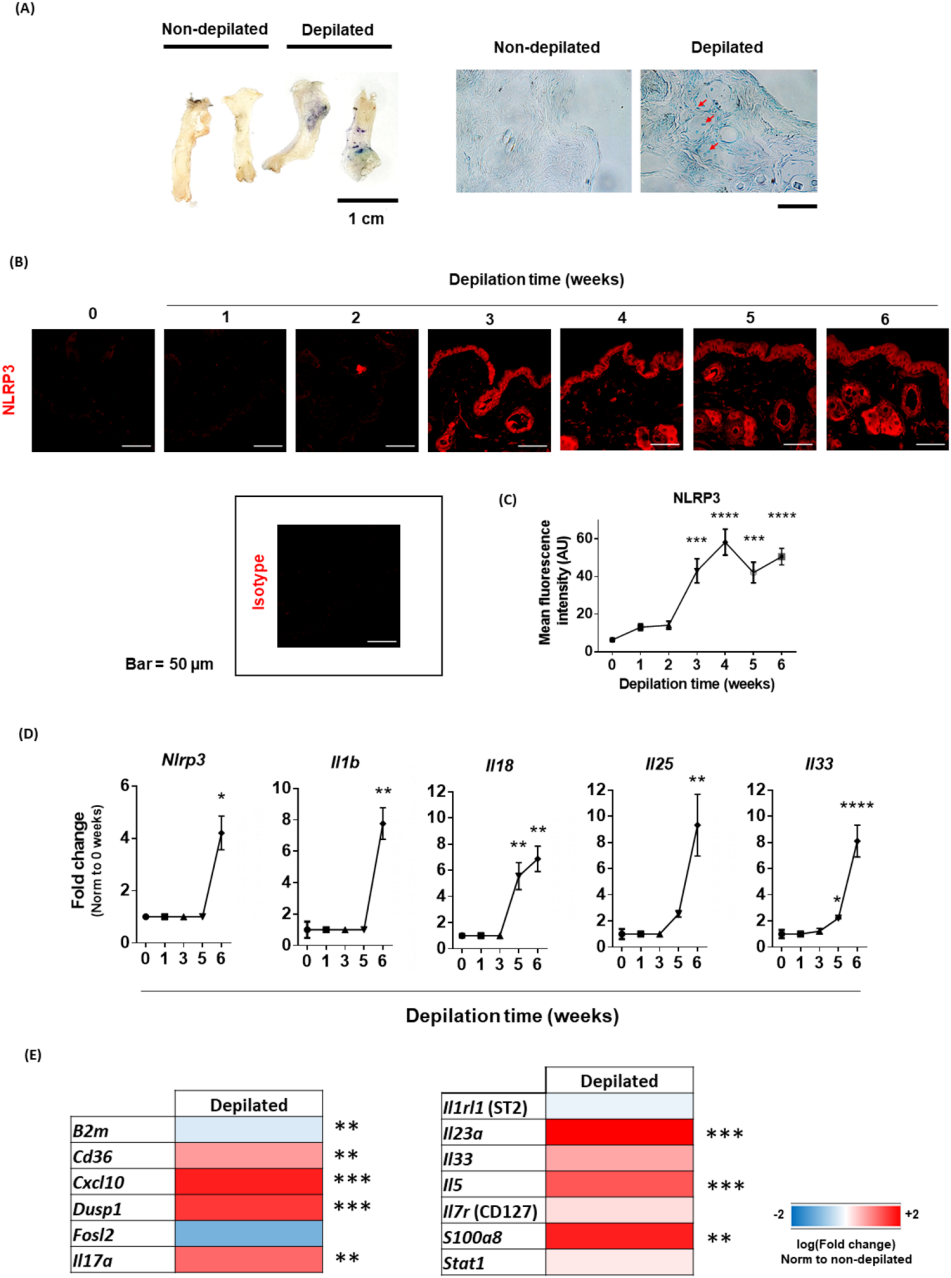
Skin depilation induces epithelial permeability and inflammasome priming in vivo. (**A**) Blue toluidine absorption in vivo by skins from non-depilated (n = 4) and depilated (n = 6) mice. The images show the opposite face where the staining was added. Representative images from confocal microscopy (×63 magnification) are also shown, remarking the staining uptake by the skin in the case of depilated specimens. Bar = 40 µm. (**B**,**C**) Kinetics and quantification of NLRP3 expression (red) in non- (0) and depilated (1–6) mice. 546-labelled anti-rabbit IgG was used as an isotype control. Three individual sections from each mouse (n = 5 animals/group and time point) were separately stained. NLRP3 expression in the whole tissue was then analyzed with a Zeiss LSM 880 confocal microscope, with two representative but distant regions from each section being chosen for quantification purposes. Data are presented as representative images and mean (SEM. Kruskal–Wallis test with Dunn’s correction for multiple comparisons). ***P < 0.001, ****P < 0.0001. (**D**) RTqPCR analysis showing the kinetics of transcriptional activation of *Nlrp3, Il1b, Il18* and alarmin genes (*Il25, Il33*) in skin biopsies from non-depilated (0) versus depilated (1, 3, 5 and 6) mice (n = 5/group, each sample analyzed in triplicates). Data are presented as mean (SEM. Mann–Whitney test). *P < 0.05, **P < 0.01, ****P < 0.0001. (**E**) Heat map of RTqPCR analyses from skin biopsies from depilated (n = 4) vs non-depilated (n = 4) mice. Each sample was analyzed in two technical replicates. In all cases, mouse models were performed in two independent set of animals. Data are presented as mean (SEM. Mann–Whitney test). **P < 0.01, ***P < 0.001.

To study the role of depilation as a first hit in allergic sensitization, first we compared the permeability of the skin barrier between depilated and non-depilated mice. As shown in Fig. 1A, blue toluidine uptake was greater in depilated specimens than in non-depilated ones, suggesting that depilation induces epithelial permeability in vivo. This result might suggest that lower layers from depilated skins are more exposed to ambient stimulus, including allergens, than untreated ones. This feature was not strain-dependent and was maintained through time, after 3 and 6 weeks of continuous depilation (Supplementary Fig. 1).

Additionally, we found that keratinocytes from depilated rodents showed a significant increase in the expression of NLRP3 protein, when compared to non-depilated animals (Fig. 1B,C). Regarding the kinetics of this protein expression, we observed that NLRP3 protein detection remained unaltered during the first 2 weeks after depilation. After the third week (Fig. 1B,C t = 3), however, the mean fluorescence intensity in the epidermis associated with NLRP3 detection was significantly enhanced when compared to non-depilated (Fig. 1B,C t = 0) specimens, remaining constant over time at least up to the last week studied in this report (Fig. 1B,C t = 6 weeks).

However, as seen in Fig. 1D, expression of *Nlrp3* transcripts was significantly upregulated when compared to non-depilated skins only after 6 weeks of depilation with the cream. This could indicate that the increase in protein detection we are observing after the third week of depilation is not due to an actual increase in the translation ratios of the protein, but rather to the formation of NLRP3 complexes, pointing to an effective priming of the pathway. Nevertheless, further studies should be conducted in order to fully validate this theory. Regarding the inflammasome products *Il1b* and *Il18*, their induction over time seems to be slightly different, with *Il18* transcripts being detected earlier (after 5 weeks) during the depilation protocol, while *Il1b* is not detected until the sixth week. A small induction of alarmins (*Il25*, *Il33*) transcription seems to be detected after 5 weeks of depilation, although this tendency was only statistically significant for the latter. However, the strongest induction was observed again after the sixth week of the protocol. When we tried to correlate the levels of *Il1b* and *Il33* transcripts with their corresponding protein counterparts, we were unable to detect them by means of Western blot and ELISA, respectively (data not shown).

Finally, after 6 weeks, skin depilation also significantly upregulated several genes related to the establishment of T1 and T3 immune responses (*Cxcl10*, *Il17a*, *Il23a*, *S100a8*) and oxidative stress (*Dusp1*, Fig. 1E), thus confirming the establishment of a proinflammatory genetic landscape when this treatment is applied long-term. Altogether, these results suggest that skin damage must be sustained over time in order to clearly induce transcriptomic changes in the tissue related to a proinflammatory background, thus validating the safety of depilatory creams when not applied regularly over healthy skins. However, the induction of protein expression due to continuous depilation over time could not be demonstrated with the studies here presented, although other depilation protocols (such as tape-stripping) have shown to induce the production of IL33 in the skin of mice^9^.

#### Thioglycolate reproduces the inflammasome activation on in vitro cultured human epidermal keratinocytes

**Figure 2:**
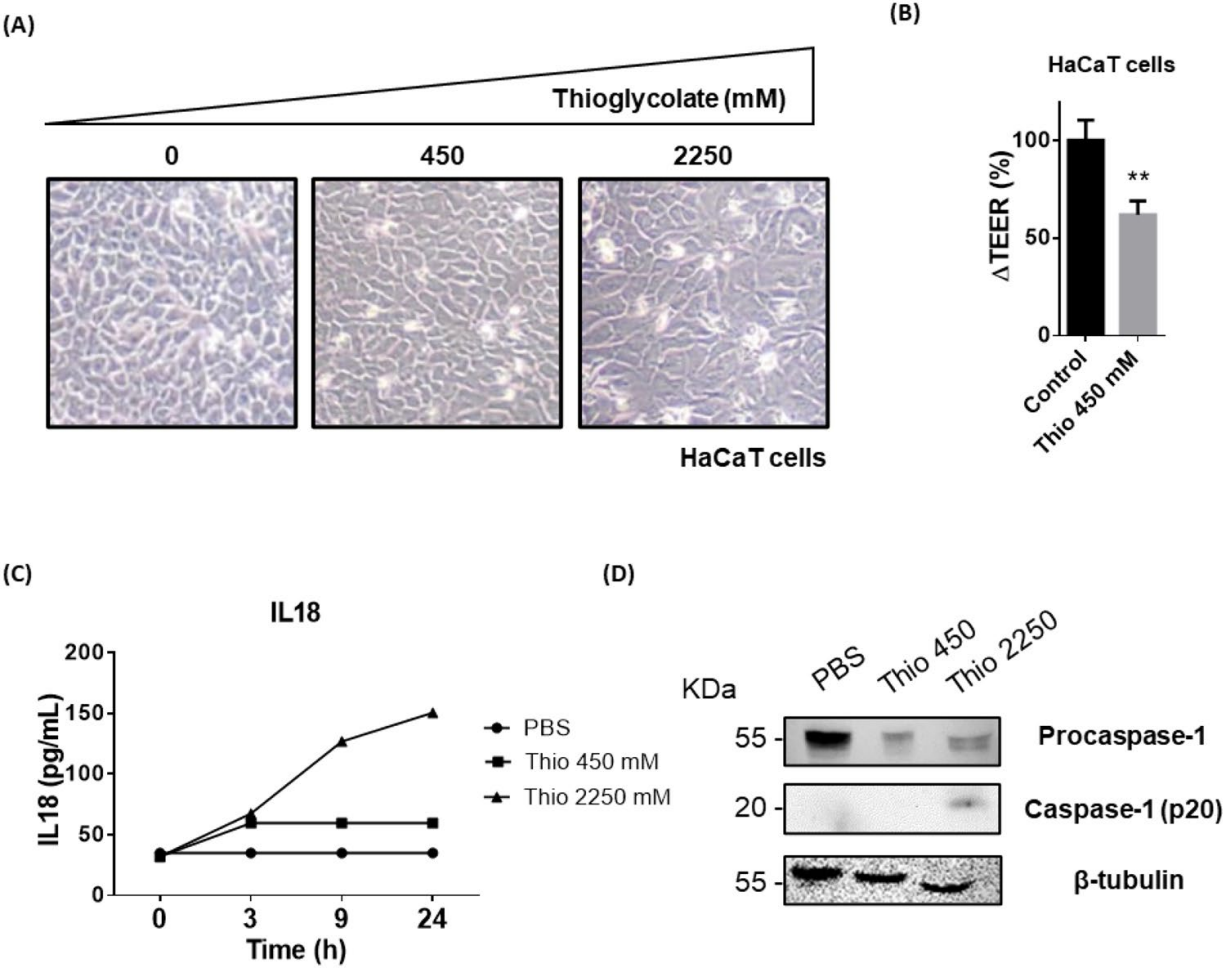
Thioglycolate induces inflammasome activation in vitro in HaCaT cells. (**A**) Dose-dependent effect of thioglycolate addition over monolayer integrity of HaCaT cells cultured in 24-well plates (n = 6/group). (**B**) Comparison of TEER values from HaCaT cells in Transwell, when treated or not with thioglycolate (450 mM). Data are presented as mean (SEM. Mann–Whitney test). **P < 0.01. (**C**) Dose-dependent effect of thioglycolate addition over the production of IL18 in HaCaT cells, as quantified by ELISA. All the experiments were performed at least three times and analyzed in triplicates. (**D**) Immunodetection of bioactive caspase-1 (p20) in lysates from HaCaT cells treated with different concentrations of thioglycolate. The experiment was performed using a pool from three biological replicates performed independently. β-tubulin was used as a loading control.

In order to furtherly validate the results obtained in mice, we sought to study the effects of the first hit (depilation) over cells from human origin. We chose thioglycolate exposure as a model of in vitro depilation because it constitutes a universal component of depilatory cream formulations. Also, previous reports had pointed that keratinocytes in the presence of this compound express an altered morphology and significantly higher levels of the stress-related chaperone Hsp70^30^. Thus, we cultured human keratinocytes (HaCaT cell line) with growing concentrations of thioglycolate. When cultured in 24-well plates, it seemed that HaCaT cells were unaffected by the addition of the stimulus, even at greater concentrations (Fig. 2A). However, when the same cells were polarized in Transwell format, the addition of thioglycolate significantly reduced HaCaT barrier capacity to 50% when compared to control conditions (Fig. 2B), according to transepithelial electrical resistance (TEER) measurement as an indicative of epidermal barrier watertightness^31^. It has been established that TEER is directly related to the number of tight junctions between epidermal cells^32^, so we hypothesize that this reduction could be a consequence of tight junction impairment.

Moreover, thioglycolate exposure prompted the release of soluble IL18 to the media, in a dose-dependent manner (Fig. 2C). Analyzing the kinetics of its production, we found that both 450 mM and 2250 mM concentrations of thioglycolate induced a small IL18 liberation 3 h after treatment; however, only with the highest thioglycolate concentration the amount of IL18 in the supernatant increased over time (Fig. 2C). Surprisingly, the addition of thioglycolate was not able to induce IL1β release to the media, with its basal concentration remaining unaffected by the stimulus after 3 h from its addition (Supplementary Fig. 2A).

In addition, we confirmed the detection of bioactive caspase-1 (p20) in cell lysates from HaCaT cells cultured with thioglycolate (2250 mM), in contrast with its absence in the same cells cultured at low concentrations (450 mM) of the stimulus (Fig. 2D). Caspase-1 is needed for both IL1β and IL18 production. Thus, the presence of the latter in cell supernatants is furtherly validated, while the absence of the first suggests an IL1β-independent inflammation mechanism. It is known that pro-IL18 is constitutively produced by epithelial cells, while translation of pro-IL1β must be activated prior to its cleavage by caspase enzymes^33^. Given the fact that we have not been able to detect pro-IL1β in skins from depilated mice, this might suggest that, although depilation protocols (or in this case, thioglycolate exposure) might not induce the translation of inflammasome-related products, priming of the NLRP3 pathway and concomitant activation of caspase-1 might be sufficient to prompt the release of bioactive IL18 from the cells, derived from the processing of the preexisting pool of pro-IL18 present on them.

In order to validate these data and its reproducibility in other human epidermis cell lines, we sought to complement the inflammasome activation study using A431 cells as another model. In this case, we observed that monolayer integrity was unaffected at concentrations used in approved formulations (450 mM), but it was compromised at higher concentrations (Supplementary Fig. 2B). Accordingly, the *Il1b* and *Il18* expression was slightly upregulated (Supplementary Fig. 2C,D) not only at a transcriptional level but also with IL18 present in cell supernatants, in a dose-dependent manner (Supplementary Fig. 2E). In the case of IL1β, we only observed small quantities of it when cells were stimulated with 2250 mM of thioglycolate, but not at lower concentrations (data not shown). Thus, these assays confirm that the results obtained with the HaCaT model are not dependent of cell line and might be extended to other human epidermis models.

### Second hit: effects of allergen encounter (Prup3_complex)

#### Allergen addition after depilation exacerbates skin inflammation in mice

**Figure 3:**
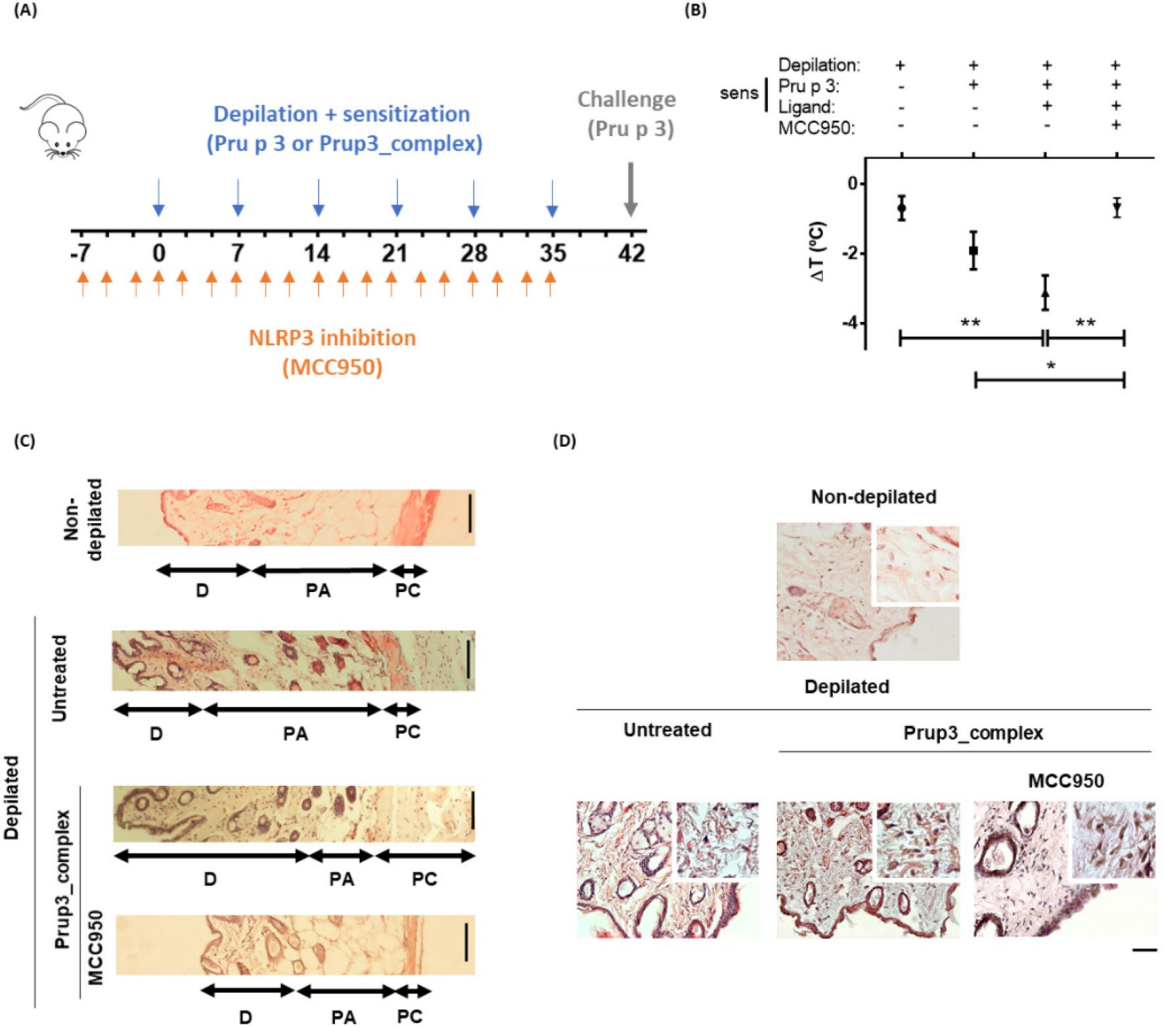
The skin pathway can induce anaphylaxis in a mouse model of allergy. (**A**) Mice were epicutaneously sensitized in the abdomen weekly (6 weeks) after depilation with Pru p 3 or Prup3_complex (n = 8/group). Sensitization with Prup3_complex was also performed in mice without functional NLRP3 (MCC950; n = 5). Untreated depilated and non-depilated animals were also included in the experimental design (n = 5/group). Challenge was performed intraperitoneally in all groups studied with 100 µg of Pru p 3 at week 7. (**B**) Comparison of body temperature drop post-challenge by sensitizing agent (n = 8 in Pru p 3 and Prup3_complex specimens; n = 5 in depilated-only and MCC950-treated animals). Data are presented as mean (SEM. Mann–Whitney test). *P < 0.05, **P < 0.01. (**C**) H&E staining of skin sections from sensitized (Prup3_complex, n = 8 mice), untreated depilated (n = 5), non-depilated (n = 5) and sensitized + MCC950-treated (n = 5) mice at ×10 and (**D**) ×40 magnification. Three individual sections from each mouse were separately stained and photographed with a Zeiss LSM 880 confocal microscope, with two representative but distant regions from each section being analyzed. After that, representative images were chosen for publication. D = dermis, PA = *panniculus adiposus*, PC = *panniculus carnosus*. Bar = 100 µm (×10) or 50 µm (×40). In all cases, mouse models were performed in two independent set of animals.

Once we have understood some of the effects derived from depilation (first hit) in skin-mediated allergic sensitization, we sought to evaluate the effects of allergen encounter (second hit) over the stressed mucosa. Pru p 3 was used as a model allergen in an adjuvant-free mouse model of anaphylaxis (Fig. 3A), based on previously published reports^10,34^. When comparing the efficiency to induce anaphylaxis of Pru p 3 vs Prup3_complex through the cutaneous route, mice sensitized with Prup3_complex showed a significant drop in body temperature compared to challenged depilated non-sensitized (Untreated) mice (Fig. 3B). In the case of Pru p 3-sensitized mice, the temperature drop, although present, was milder than Prup3_complex-sensitized specimens (Fig. 3B).

In order to demonstrate the importance of the first hit (depilation and NLRP3 priming) to establish the allergic phenotype, mice sensitized with Prup3_complex were treated with MCC950. This compound has been extensively used as an inhibitor of NLRP3 oligomerization^35^, consequently interrupting the activation of the inflammasome pathway and the release of its associated cytokines. Remarkably, mice sensitized with Prup3_complex which lacked a functional NLRP3 pathway did not suffer from anaphylaxis after being challenged with Pru p 3, and their drop in body temperature was significantly milder even when compared to mice with functional NLRP3 that had been sensitized with only Pru p 3 (Fig. 3B). Consequently, we concluded that activation of the inflammasome pathway in stressed skin prior to allergen encounter is crucial to initiate an allergic sensitization response in the tissue that can translate into a systemic reaction in the individual. Surprisingly, we were not able to detect allergen specific sIgE and sIgG1 in our model by means of ELISA (Supplementary Fig. 3) that justifies the observed anaphylactic reactions. This might be due to limitations of the strain used in these experiments (BALB/c), since Morafo et al*.* demonstrated that different mouse strains have different susceptibilities to generate sIgE antibodies against allergens, in a food-dependent manner. For example, BALB/c mice are not well responders to cow milk, while they develop good sIgE titers against peanut^36^.

Besides, histological characterization of Prup3_complex sensitized mice revealed remodeling of skins, with thickened dermis and striated muscle layers compared to depilated non-sensitized specimens, as well as non-depilated ones (Fig. 3C). Nevertheless, NLRP3 priming due to first hit seems crucial to allow the remodeling induced by Prup3_complex, since specimens sensitized with the allergen and treated with MCC950 presented a phenotype more similar to both depilated non-sensitized specimens, as well as non-depilated ones (Fig. 3C). Additionally, cellular infiltration into the dermis was higher in sensitized animals, suggesting that Prup3_complex exposure prompts cellular mobilization in the mucosa (Fig. 3D). All these images were taken after the mice were challenged with Pru p 3, although the possibility that the observed changes are due to the challenge is ruled out, since untreated and non-depilated specimens did also receive the same challenge. So, these results might point that Prup3_complex addition after depilation aggravates the skin inflammation previously observed in depilated only mice (Fig. 1).

#### Prup3_complex exposure induces cellular infiltration across different mucosal locations

**Figure 4:**
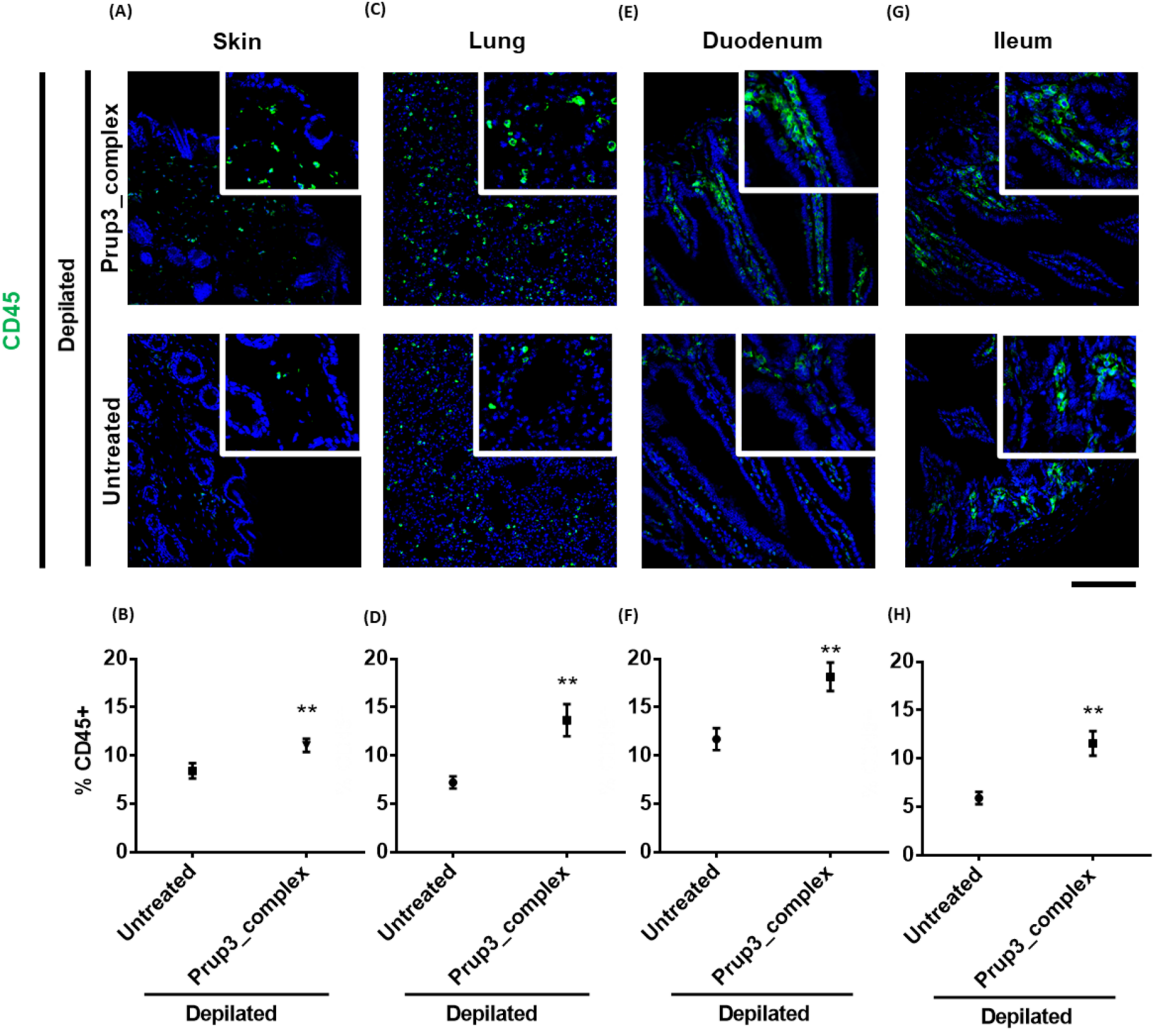
CD45+ infiltration in mucosa after Prup3_complex cutaneous treatment. Representative images and quantification of (**A**,**B**) skins, (**C**,**D**) lungs, (**E**,**F**) duodena and (**G**,**H**) ilea hybridized with anti-CD45 antibody (green). Nuclei (blue) stained with DAPI. Quantification of CD45+ cell infiltration was calculated as the ratio between CD45+ and DAPI+ cells per image (n = 5/group; at least 3 sections were separately stained from each mouse at distal depths of the tissue and 3–5 images were taken per section). Data are presented as mean (SEM. Mann–Whitney test). **P < 0.01. Bar = 100 µm. In all cases, mouse models correspond to samples from two independent sets of animals.

Given these results, we sought to analyze if allergen encounter could induce modifications in the immunological state of depilated skins. As shown in Fig. 4A,B, we found that Prup3_complex addition induced a significant CD45+ infiltration in the skin of depilated mice. Interestingly, this recruitment of leukocytes was also observed in distant mucosal locations, such as lungs, duodena, and ilea (Fig. 4C–H). To frame the contribution of depilation or Pru p 3 (without its ligand) in the recruitment of immune cells, we also analyzed non-depilated mice, as well as depilated Pru p 3-sensitized animals. As shown in Supplementary Fig. 4, non-depilated and depilated mice present similar levels of cell infiltration in the dermis. Also, Pru p 3 presents slightly greater levels of CD45+ cells in the region, although Prup3_complex is the only treatment presenting significant differences compared to non- and depilated animals. So, immune cell recruitment results from allergen encounter (second hit) during sensitization, with the ligand of Pru p 3 playing an important role in this process. As in the case of Fig. 3C,D, all these images were taken after the mice were challenged with Pru p 3, but the possibility that the observed changes are due to challenge is ruled out, since non-depilated, depilated and Pru p 3-sensitized specimens did also receive the same challenge, but their infiltration is still below Prup3_complex-sensitized mice’s levels.

#### Infiltration in sensitized skins includes ILC-resembling lymphocytes

**Figure 5:**
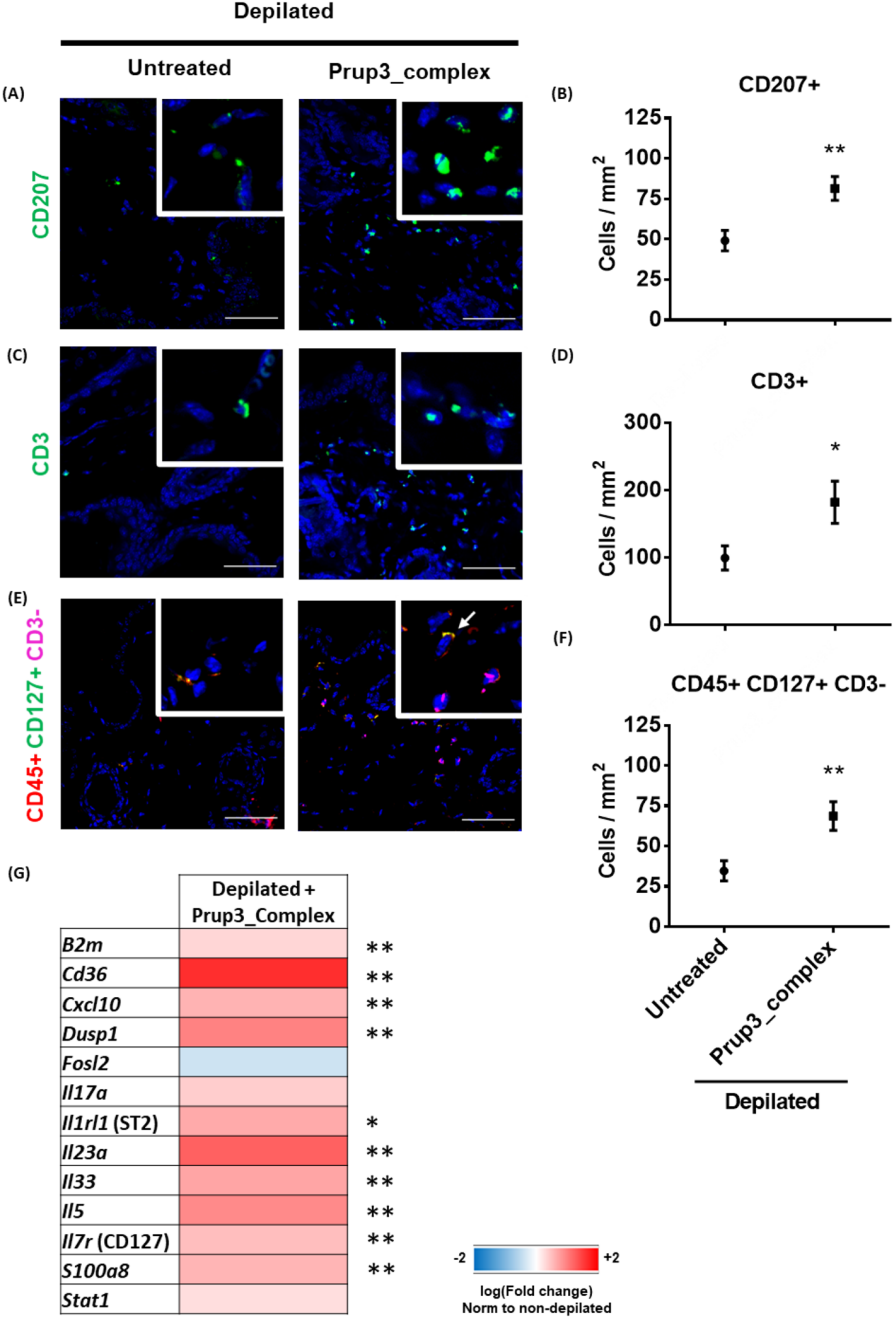
Prup3_complex prompts inflammatory phenotype in the skin. Representative images and quantification of immune infiltration in the skin of depilated sensitized (Prup3_complex; n = 5) and untreated (Untreated; n = 5) mice. Nuclei (blue) stained with DAPI. (**A**,**B**) CD207+ (DCs), (**C**,**D**) CD3+ (T cells) and (**E**,**F**) CD45+CD127+CD3− (ILCs) cells. Quantification of DCs and ILCs is referred to dermal areas where these cells were grouped forming clusters (at least 4 sections were obtained from each mouse at distal depths of the tissue and 3–5 images were taken per section), while T cells were homogeneously distributed through the whole tissue. Data are presented as mean (SEM. Mann–Whitney test). *P < 0.05, **P < 0.01. Bar = 50 µm. (**G**) Heat map of RTqPCR analyses from skin biopsies from depilated + Prup3_complex (n = 2) vs non-depilated (n = 4) mice. Each sample was analyzed in two technical replicates. Data are presented as mean (SEM. Mann–Whitney test). *P < 0.05, **P < 0.01. In all cases, mouse models correspond to samples from two independent sets of animals.

To characterize the nature of the allergen-induced infiltration, different cell types were stained with specific cell markers in skin biopsies of Prup3_complex sensitized mice. We observed that the CD45+ infiltration included mainly dermal CD207+ (Fig. 5A,B), CD3+ (Fig. 5C,D) and CD127+CD3− cells (Fig. 5E,F; zoomed in Supplementary Fig. 5), the later ones resembling an ILC-like phenotype^37^. In fact, the transcriptional analysis of sensitized skins revealed that several genes related to ILC2 biology were significantly upregulated in mice sensitized with Prup3_complex, when compared to non-depilated animals, including *Cd36* (which is required for ILC2 activation through a PPARɣ-dependent mechanism)^38^, *Il1rl1* (the receptor for IL33), *Il33* and *Il7r* (CD127) (Fig. 5G). Thus, transcriptomic results support the idea that these cells might be indeed from ILC origin. However, which one of these subsets (DCs, T lymphocytes or ILCs) is the key contributor towards the pro-allergic response orchestrated by Prup3_complex in murine skins has not been studied and should be furtherly discussed in future publications.

### In vitro responses of ILC2s from healthy volunteers and peach allergic patients

#### Prup3_complex induce phenotype switch in human ILC2s

**Figure 6:**
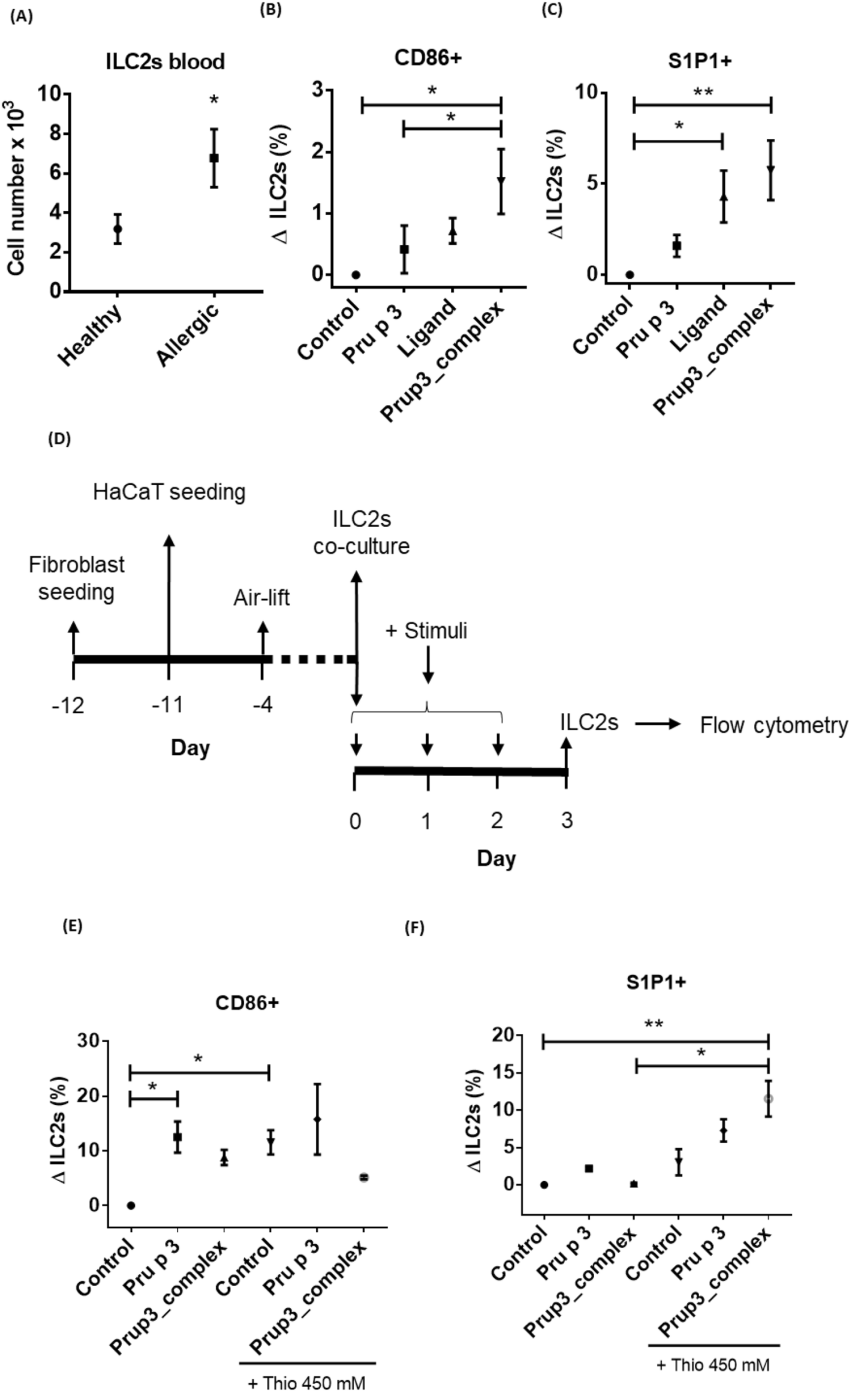
Prup3_complex induces phenotype changes in peripheral human ILC2s in vitro. (**A**) Quantification of ILC2s in the bloodstream of allergic (LTP+ ; n = 9) and healthy (n = 9) donors. Total cell counts were determined with a hemocytometer. Data are presented as mean (SEM. Mann–Whitney test). *P < 0.05. (**B**,**C**) Flow cytometry phenotyping of circulating ILC2s incubated for 96 h with Pru p 3, Pru p 3’s ligand or Prup3_complex. The experiment was repeated 5 independent times in duplicates. Data are presented as a mean of ∆ILC2 = (% Positive ILC2s)_sample_ – (% Positive ILC2s)_control_ (SEM. Kruskal–Wallis test with Dunn’s correction for multiple comparisons). *P < 0.05, **P < 0.01. (**D**) Work diagram from the 3D in vitro skin model. (**E**,**F**) Flow cytometry as in (**B**,**C**).

Given the relevance that ILC2s have gained over the last decades in the study of allergic pathologies^21^, we decided to launch a low-scale study of the potential effects of Prup3_complex over ILC2s, independent from the murine model reported before in this manuscript. First, we isolated these cells from human blood samples, and we found that patients allergic to peach showed significantly increased numbers of circulating ILC2s when compared to healthy controls (Fig. 6A). Then, they were cultured with Pru p 3, the lipid ligand of Pru p 3 or Prup3_complex. Under these conditions, only those cells treated with Prup3_complex significantly overexpressed the costimulatory molecule CD86 (Fig. 6B), although this induction was very mild (+ 1.52%). Interestingly, this was accompanied by the overexpression of the sphingosine-1-phosphate receptor 1 (S1P1), which was significantly stimulated by both the lipid ligand of Pru p 3 and Prup3_complex (Fig. 6C).

To analyze a model closer to physiological conditions, we developed an in vitro skin model based on keratinocytes (HaCaT cells) and fibroblasts cultured in a Transwell format. ILC2s from healthy volunteers were seeded onto the basolateral chamber. Pru p 3 or Prup3_complex was added to the apical side in the presence or absence of thioglycolate, to replicate an in vitro sensitization process (Fig. 6D). Expression of CD86 and S1P1 in ILC2s was analyzed by flow cytometry, showing CD86 was overexpressed after incubation with both Pru p 3 and Prup3_complex, independently of thioglycolate addition (Fig. 6E). In contrast, S1P1 expression in ILC2s was only significantly induced by Prup3_complex after thioglycolate addition (Fig. 6F) suggesting that overexpression of S1P1 in ILC2s is dependent of tight junction disruption in the skin monolayer due to thioglycolate treatment (as shown in Fig. 2B), resulting in a permeability increase in the tissue and a greater availability of the allergen in the basolateral chamber after that. Nonetheless, these results should not be seen as a reflection of what is happening in murine skins, since experimental conditions from both assays differ greatly. For example, based on our experimental data, we cannot discriminate if ILCs detected in murine skins (Fig. 5E,F) are resident ILCs or migratory peripheral ones that were recruited to the tissue after allergen exposure, while in the case of human ILC2s (Fig. 6) they have been isolated in all cases from peripheral circulation. For future publications, it would be interesting to isolate the dermal ILC-like subset detected in Prup3_complex-sensitized mice, to analyze if its phenotype has also been switched (when compared to non-sensitized controls) in a similar fashion of human ILC2s do when they get in contact with the allergen.

## Discussion

Based on the two-hits hypothesis to explain the immune mechanism of several chronic inflammatory diseases, allergy could be the result of: first, skin depilation, which primes the inflammasome pathway; and second, allergen encounter, which activates cell infiltration and systematizes the response. In this work, we have deepened in the mechanisms by which depilated skin can prime allergic sensitization to food allergens. We produced an epicutaneously-sensitized murine model of allergy that showed significant anaphylactic symptoms after allergen challenge, accompanied by skin remodeling, which included higher levels of cellular infiltration in the dermal layer, a histopathological feature frequently reported in atopic dermatitis^39^. The skin has been described as an important route of sensitization^8–10^ and there is a high prevalence of IgE antibodies to foods in atopic dermatitis, a chronic pruritic inflammatory skin disease^40^. Additionally, epidemiological evidence show that environmental exposure to allergenic proteins leads to the development of food allergy in infants with impaired skin barriers^41^.

Even though skin has been classically described as a tolerogenic tissue^42,43^, there is growing evidence that environmental stressors can reverse this tolerogenic environment and elicit T2 responses to promote food allergy sensitization^44^. Our findings demonstrate that depilation augments skin permeability and the production of the inflammasome receptor NLRP3 (in vivo, murine keratinocytes), as well as the proinflammatory cytokine IL18 (in vitro, HaCaT and A431 human cell lines). This is supported by the fact that cleaved caspase-1 (20 kDa) was found in HaCaT cell lysates incubated with high doses of thioglycolate, one of the main compounds of depilatory creams. Altogether, these features suggest that depilation primes the NLRP3 pathway in the skin, playing as a “first hit” of inflammation that breaks the tolerogenic environment of the tissue^45^.

Additionally, depilation prompted the upregulation of *Il25* and *Il33* transcripts in vivo, suggesting a possible relationship with the initiation of T2 responses in the tissue, although detection of these proteins in murine skins cannot be confirmed. The importance of NLRP3 priming to the promotion of allergic sensitization is supported by the fact that mice treated with MCC950 (i.e., lacking a functional NLRP3 route) are unable to develop a strong anaphylactic response against Prup3_complex (Fig. 3B). This result is in accordance with recent reports in the literature which demonstrate that inflammasome activation due to respiratory syncytial virus infection can lead to asthma development in BALB/c mice^46^. Furthermore, cross-sectional studies with obese asthmatic patients have shown the correlation between NLRP3 activation and higher IL5 levels, leading to worse symptomatology of the disease^47^. Finally, it has been recently reviewed that innate pathways in mucosal sites might be driving tolerance breakdown and food allergy sensitization, although specific mechanisms remained unknown up to now^48^. Thus, this manuscript offers a preliminary study about the potential consequences that skin depilation might have over food allergy development, opening some research lines about the molecular pathways connecting both events that could be discussed in future publications.

Regarding the effects of the “second hit” (allergen encounter), we found that cutaneous exposure to Prup3_complex induces an immune response based on the recruitment of CD45+ cells to the exposed tissue. This immune infiltration is exclusively a result of allergen presence, since depilated untreated animals present CD45+ infiltration levels analogous to non-depilated control mice. Moreover, we also have shown that this infiltration is not limited to the skin but spread through different mucosal locations. Systemization of immune reactions initiated locally in the skin has been reported not only in food allergy^9,11^ but also in defense responses against *Staphylococcus aureus*, proving that cutaneous exposure to pathogenic bacteria ameliorate the symptomatology of secondary infections in the lung through an IgE- and mastocyte-mediated mechanism^49^. In our study, leukocyte infiltration was also detected in lungs, duodena and ilea of anaphylactic mice sensitized through this route. A deeper characterization revealed that the cutaneous infiltration was mainly due to dermal DCs, T cells and ILCs. Hence, allergen encounter would act as the “second hit” in the two-hit model of allergic sensitization, prompting mobilization of immune cells towards the skin tissue. However, based on our data we cannot discriminate the contribution of each cellular type to the establishment of the allergic response in vivo.

On the other hand, when we isolated ILC2s from peripheral blood of patients allergic to Pru p 3, we observed that they present significantly higher levels of these cells in circulation than healthy control donors. This observation is in accordance with previous reports of peripheral ILC2s growing in number after intranasal challenge and during pollinic season in cat and *Phleum pratense* allergic patients, respectively^50,51^. Also, our in vitro skin model of allergic sensitization showed that, after allergen exposure in the epithelium, ILC2s overexpressed CD86, confirming that exposure to Prup3_complex can modify their phenotype. Interestingly, the level of overexpression of CD86 was greater in ILC2s cultured in the skin model than those cultured directly with the allergen (for example, 12.5% vs 0.4% in the case of Pru p 3; Fig. 6E vs. B). These results might point to an important role of the epithelium in the induction of the phenotype variation induced by the allergen.

Moreover, in an impaired barrier by thioglycolate addition, Prup3_complex is able to induce the overexpression of the receptor S1P1 in the surface of ILC2s. Given the structural resemblance between Pru p 3 ligand and this immune mediator^27^, this might suggest that ILC2s may sense the lipid fraction of the allergen through this receptor. Supporting this observation, we have recently reported that, in an in vitro model of human skin, keratinocytes are able to metabolize Pru p 3 ligand into a derivative of S1P, which is able to induce the migration of monocytes as efficiently as this chemokine^29^. Summing up, these in vitro results point to the fact that both signals (skin abrasion and allergen encounter) act synergistically to induce phenotype changes in peripheral ILC2s, although immunological consequences of this phenotype switch and its direct implications in allergy sensitization have not been studied in this work and, thus, they should be addressed in future publications. In addition, an important limitation of this study is that due to their low numbers these results could not be analyzed directly in murine skin ILC2s, but rather in human peripheral ones. Thus, it would be interesting to launch a large-scale model of skin-mediated allergic sensitization that would allow to discuss if all the changes induced by Prup3_complex in peripheral ILC2s can be replicated in situ in the sensitized tissue.

**Figure 7:**
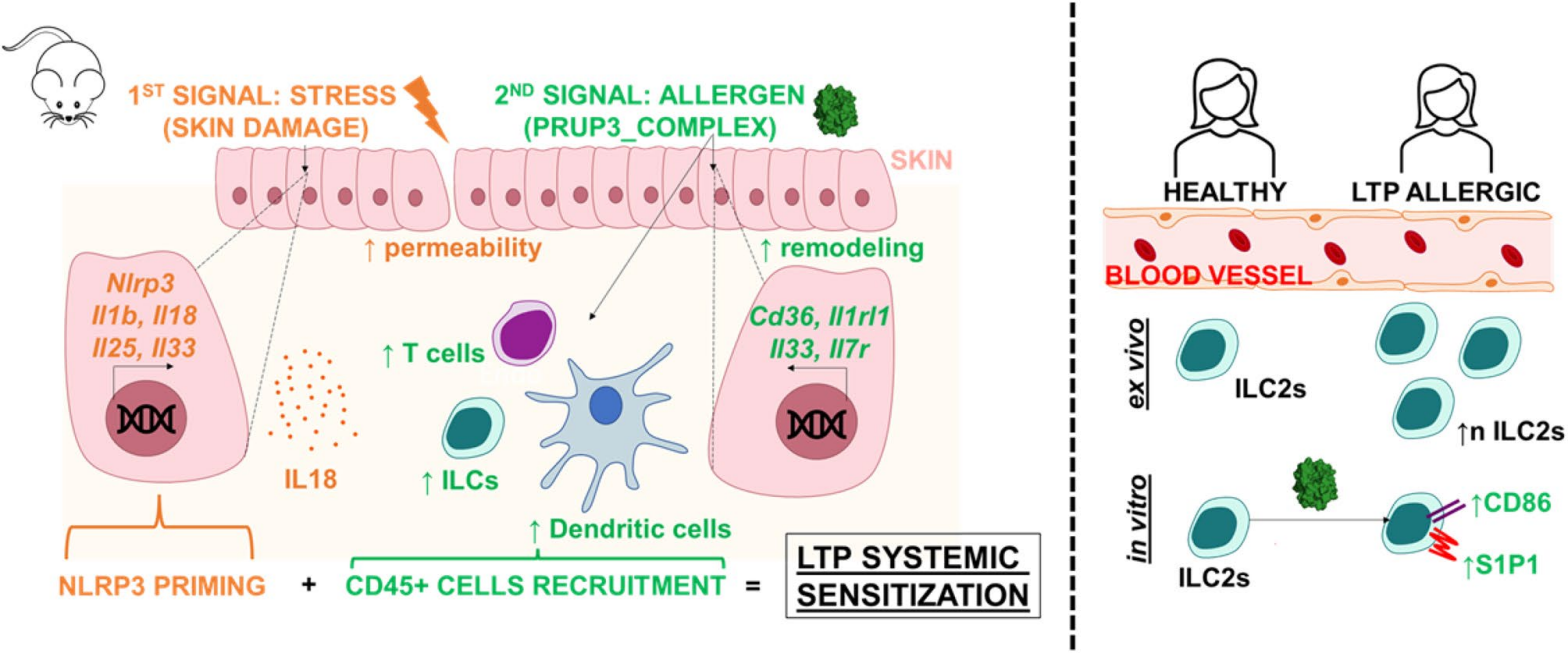
Proposed model for skin-mediated allergy sensitization and observations made with human ILC2s.

Considering all these observations, the following model is proposed to explain a possible timeline of allergic sensitization. As shown in Fig. 7, allergen sensitization through the skin would require two necessary hits: (1) first, a stressing agent, that primes the inflammasome pathway in keratinocytes, as well as the production of alarmins; and (2) the second signal, the presence of an allergen, which directs the proinflammatory response induced by the abrasion towards a T2-skewed proallergic response, promoting increased numbers of local DCs, T cells and ILCs. Furtherly, data derived from in vitro cultured human ILC2s suggest that certain allergens, such as Prup3_complex, can induce a phenotype change in these cells, although implications of these changes in food allergy development need further research to be outlined.

## Methods

### Allergen samples

Recombinant Pru p 3 was produced in *Pichia pastoris* as previously described^52^ and incubated with its ligand in a 10:1 ratio, thus forming “Prup3_complex”^10^. Its purity was assessed by mass spectrometry. In addition, the amount of LPS was quantified using Thermo Scientific’s Pierce Chromogenic Endotoxin Quant Kit. Samples with values above 0.01 EU/mL were discarded. The ligand of Pru p 3 was chemically synthesized as previously reported^29^, by incubating 10-hydroxycamptothecin (Sigma-Aldrich, Germany) and phytosphingosine (Sigma-Aldrich) O/N at 4 °C in 50 mM MES buffer (2-ethanesulfonic acid, Sigma-Aldrich) 0.01% Tween 20 (Sigma-Aldrich) 0.25 mM N-hydroxysuccinimide (NHS, Sigma-Aldrich) 0.1 mM 1-ethyl-3-(2-dimethylaminopropyl)-carbodiimide (EDC, Sigma-Aldrich).

### Animal models

For the skin permeability assay, 24-week-old female C57BL/6J mice (Charles River, L’Arbresle, France) were anesthetized by inhalation of isoflurane and abdominal fur was removed just one time with depilatory cream (Depilated) or scissors, without damaging the skin (Non-depilated). Immediately after, the abdomen was briefly washed with warm water and 40 µL of blue toluidine solution 0.1% (Sigma) were added in small droplets in the region. After 5 min, the staining was removed with a dry gauze and the animals were euthanized using a CO_2_ chamber. Abdominal skin was removed, and tissues were photographed prior to its inclusion in paraffin. Once they were included, 7 µm sections were cut using a microtome, deparaffined with xylene and decreasing concentrations of ethanol and mounted with ProLong Gold Antifade Mountant (Thermo Fisher) without further staining, followed by immediate visualization with a Zeiss LSM 880 confocal microscope (63× magnification). To discard strain-dependent conclusions and the maintenance of the results through time, the same protocol was performed in female BALB/c mice at different time points (Supplementary Fig. 1). In this case, the photographs were taken after the skins were frozen in liquid N_2_.

For the effects of depilation over inflammasome priming, 6–8-week-old female BALB/c mice (Charles River) were weekly depilated once per week (for 6 weeks) in the abdomen with depilatory cream and euthanized with CO_2_ at different time points. Non-depilated age- and sex-matched specimens were used as controls. Abdominal skin samples were collected and included in paraffin for immunofluorescence and histological analyses.

For the effects of allergen encounter over cellular infiltration and RTqPCR analyses, 6–8-week-old female BALB/c mice were weekly sensitized once per week (for 6 weeks) epicutaneously with the allergen (100 µg of Pru p 3 or Prup3_complex; Fig. 3A) after depilation. The depilation protocol we followed was analogous to the one described in the prior paragraph. One week after the last sensitization, mice were provoked with an intraperitoneal injection of 100 µg of Pru p 3. Body temperature was measured before and 15 min after challenge by rectal thermometer (VWR). Mice were euthanized in a CO_2_ chamber immediately after this measure and tissue samples were collected, after perfusion with PBS. Age- and sex-matched animals were also depilated, but non-sensitized, and challenged under the same conditions. Where indicated, age- and sex-matched control specimens were non-depilated nor-sensitized prior to being intraperitoneally challenged. For histological analyses, skin, lung, and gut biopsies were included in paraffin after euthanasia with CO_2_.

In order to evaluate the relevance of NLRP3 priming towards the induction of allergic sensitization, we repeated the sensitization with Prup3_complex in BALB/c mice, but 1 week prior to the first sensitization protocol (days − 7 to 0) we started to treat them intraperitoneally as previously reported^53^ with the inhibitor MCC950 (10 µg/g). As shown in Fig. 3A, the inhibition was performed 3 times per week and was continued through the sensitization protocol (days 0 to 35), but not before the intraperitoneal challenge with Pru p 3 was applied (day 42).

All animals were randomly assigned and separated into each group immediately after its arrival to our facilities. They were fed ad libitum with a Pru p 3-free diet (Labdiet Eurodent Diet 22% pellet for rodents). All the procedures were carried at the IIS- Fundación Jiménez Díaz (FJD, Madrid, Spain) with the permission of the Institutional Animal Care and Use Committee from the Community of Madrid (Ref. PROEX 392/15), under the current legislation (European Union Directive 2010/63/EU) and in compliance to ARRIVE guidelines.

### Histological analyses

Sections (7 µm) of the paraffin-embedded organs were cut using a microtome (Leica RM1235, Wetzlar, Germany). After deparaffination and rehydration, hematoxylin/eosin (H&E) staining was performed following provider’s instructions (Sigma-Aldrich, St. Louis, MO, USA) and samples were mounted using DPX (Sigma-Aldrich). Immunofluorescence (IF) assays were performed as previously described^54^. Briefly, rehydrated tissues underwent antigen retrieval with pH = 9 Tris–EDTA Tween 0.05% for 10 min at 87 °C, subsequently being cooled down at room temperature (RT) for 15 min. After washing, samples were blocked for 1 h at RT with a mixture of PBS, 10% bovine serum albumin (BSA) and 5% fetal bovine serum (FBS). Incubation with primary antibodies was performed for 1 h at RT and, after washing, specific secondary antibodies were added. Nuclei staining was performed with DAPI and specimens were mounted with ProLong Gold Antifade Mountant (ThermoFisher). Images were obtained with a Zeiss LSM 880 confocal microscope, using 405, 488, 561 and 633 nm laser excitations. Graphical material was analyzed with ZEN 3.1 and Image-Pro Plus software. Additional information about the antibodies used for the analyses can be found in Supplementary Table 1. Isotype controls for all the analyses can be found in Supplementary Fig. 6.

### Gene expression by qPCR

mRNA was isolated from frozen skin samples using the PureLink RNA Micro Kit (ThermoFisher). mRNA samples from non-depilated (n = 4), depilated (n = 4) and depilated + sensitized (n = 2) animals were isolated, and cDNA was obtained with the High-Capacity cDNA Reverse Transcription Kit (ThermoFisher), following provider’s instructions. qPCR was performed from each individual sample using Bio-Rad’s PrimePCR Pathways (Atopic Dermatitis M384; Hercules, CA, USA), following provider’s instructions. Each assay was repeated twice. Additionally, gene expression from individually selected genes was analyzed using the FastStart Universal SYBR Green (ROX) (Roche) as previously described^29^, using the oligonucleotides detailed in Supplementary Table 2, which were designed with the help of Primer-BLAST software^55^. Relative expression of each gene was referred to those of the untreated samples. *Hprt* and *Ppia* were used as endogenous genes to normalize the results.

### In vitro keratinocytes assays

A431 cells (ATCC CRL-1555; human epidermal keratinocytes) or HaCaT cells (CVCL 0038; human epidermal keratinocytes) were seeded into 24-well plates (Costar, New York, NY, USA) and grown in supplemented DMEM. Once confluence was reached, cells were incubated with growing concentrations of sodium thioglycolate (90, 450 and 2250 mM; Sigma-Aldrich) for 5 min, followed by extensive washes with PBS. This protocol was repeated once a day for 72 h and, after that, supernatants were collected at different time points and IL1β or IL18 were quantified using Cusabio’s Human ELISA kits (Wuhan, China), following provider’s instructions. Cell culture images were obtained with an Olympus CKX31 optical microscope.

Additionally, 6 h after the last thioglycolate addition cells were lysed and homogenized with GIT extraction buffer (pH 7; 4 M guanidine isothiocyanate, Sigma-Aldrich; 25 mM sodium citrate, Sigma-Aldrich; 0.5% sarcosyl, Sigma-Aldrich; 0.1 M 2-mercaptoethanol, Carl Roth, Germany). RNA was purified by phenol:chloroform extraction and precipitated with ethanol. cDNA was obtained and qPCR was performed as previously described in the “Gene expression by qPCR” section. *Gapdh* was used as an endogenous gene to normalize the results. Alternatively, intracellular protein content was isolated by incubation for 10 min (4 °C) with 0.5 mL of RIPA buffer (20 mM Tris–HCl pH 8, 150 mM NaCl, 1% Triton X-100, 0.1% SDS) and EDTA-free protease inhibitor cocktail (Roche). After that, samples were centrifuged for 10 min (4 °C) at 10,000*g* and supernatants were collected. Equal amounts of protein were loaded into 15% SDS-PAGE gels and transferred to PVDF membranes. After blocking O/N with Casein Blocking Buffer (Sigma), Western blot was performed with an anti-caspase-1 (1:500; 22915-1-AP) antibody O/N, followed by extensive washing with PBS-Tween (0.05%) and 1 h incubation with anti-rabbit-HRP (Sigma, 1:30000). The reaction was revealed using Thermo Scientific’s Super Signal West Pico PLUS Chemiluminescent Substrate for 4 min (RT) and GE Healthcare’s Amersham Hyperfilm ECL chemiluminescence films. Uncropped images can be found in Supplementary Fig. 7.

### Isolation of ILC2s

Patients with a clear clinical history of peach allergy and healthy donors were voluntarily recruited and peripheral blood was donated at the FJD Hospital (Madrid, Spain). All individuals were included in the research after providing informed consent. All experimental protocols were conducted in accordance with the latest revision of the Declaration of Helsinki after being approved by the ethical committee from Universidad Politécnica de Madrid (LILIPAL_BIO2017-84548-R).

Peripheral blood mononuclear cells (PBMCs) from each subject were isolated using a Lymphoprep density gradient and ILC2s were purified using STEMCELL’s EasySe Human ILC2 Isolation Kit (STEMCELL Technologies, Grenoble, France), following provider’s instructions.

### ILC2s allergen stimulation

ILC2s were seeded into 96-well plates (Costar) at a concentration of 4000 cells/mL and cultured for 96 h in supplemented RPMI medium in the presence of Prup3_complex (5 ng/µL) or only Pru p 3 (5 ng/µL) or Pru p 3 ligand (0.5 ng/µL), respectively. After, cells were incubated for 1 h at RT with specific antibodies against sphingosine-1-phosphate receptor type 1 (S1P1; 1:50; FAB7089P PE-conjugated; R&D Systems) and CD86 (1:50; BU63 APC-conjugated; ImmunoTools). Samples were analyzed using a BD Accuri cytometer. Unstained samples were used as background control.

### In vitro skin model of allergic sensitization

To simulate the two-hit allergic sensitization hypothesis in vitro, we developed a 3D skin model based on 24-well Transwell plates as previously reported, with slight modifications^29^. Briefly, plates were coated with a matrix of collagen from human placenta and seeded with human fibroblasts (BJ cells; ATCC CRL-2522). After 24 h, human epidermal keratinocytes (HaCaT cells) were seeded towards the apical chamber and grown for a week with supplemented DMEM. After that, cells were cultured on airlift conditions for an additional 96 h. Finally, ILC2s were added in the basolateral chamber and the in vitro skin model was incubated with 450 mM thioglycolate for 5 min, followed by extensive washes with PBS. This protocol was repeated once a day for 72 h. After each thioglycolate hit, Pru p 3 or Prup3_complex was added at a final concentration of 5 ng/µL. To compare the sensitizing capacity of the allergens without the first hit (depilation) of the allergic sensitization process, in some cases the allergens were added following the same pattern, but without the previous addition of thioglycolate to the wells. 24 h after the last hit, ILC2s were collected and incubated for 1 h at RT with specific antibodies against S1P1 (1:50; FAB7089P PE-conjugated; R&D Systems) and CD86 (1:50; BU63 APC-conjugated; ImmunoTools). Samples were analyzed using a BD Accuri cytometer. Unstained samples were used as background control.

### Statistical analyses

Statistically significant differences were analyzed by GraphPad6 (GraphPad Software Inc., La Jolla, CA, USA) using Mann–Whitney test, except where noted. In all cases, P values < 0.05 were considered significant.

## Supplementary Information

Supplementary Information.
