# Supplementary material for: NLRP3 priming due to skin damage precedes LTP allergic sensitization in a mouse model
Source: Sci Rep. 2022 Feb 28;12:3329. doi: 10.1038/s41598-022-07421-y (PMC8885703; doi:10.1038/s41598-022-07421-y)
Supplement: Supplementary file 1 — Supplementary Information. [file 41598_2022_7421_MOESM1_ESM.pdf]

SUPPLEMENTARY FIG 1

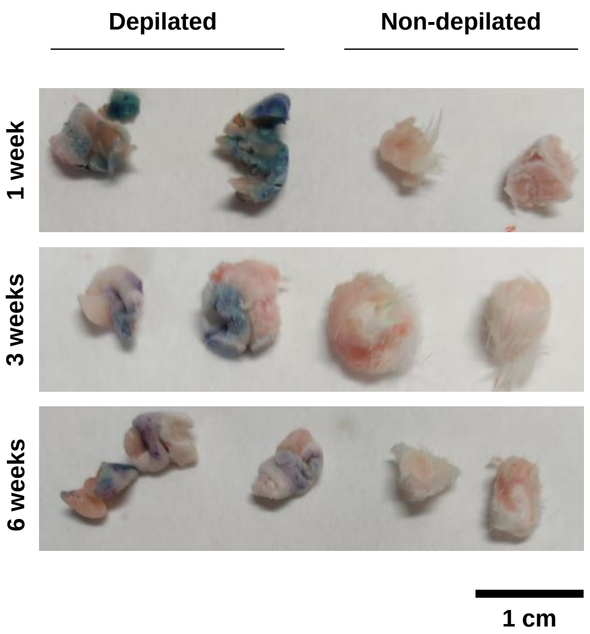

Supplementary Fig. 1. Kinetics of permeability increase due to skin depilation in murine skins. Blue toluidine absorption *in vivo* by skins from non-depilated (n = 2 / time point) and depilated once per week (n = 2 / time point) BALB/c mice.

SUPPLEMENTARY FIG 2

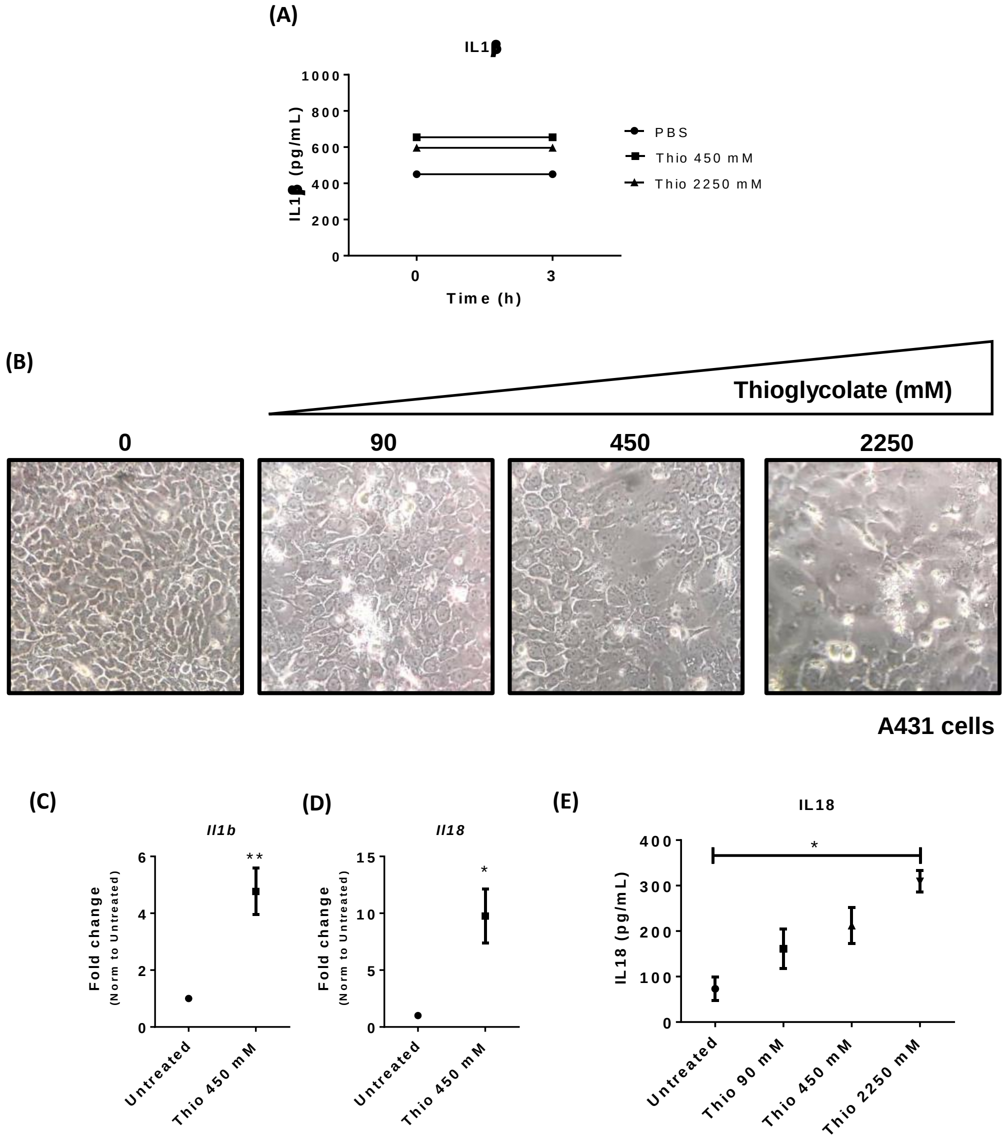

Supplementary Fig. 2. Thioglycolate induces inflammasome activation *in vitro* in A431 cells. (**A**) ELISA analysis showing no changes in IL1β detection due to thioglycolate addition after 3 h in HaCaT cells. (**B**) Dose-dependent effect of thioglycolate addition over monolayer integrity of A431 cells cultured in 24-well plates (n = 6 / group). (**C, D**) RTqPCR analysis showing the overexpression of *Il1b* and *Il18* transcripts in A431 cells treated with thioglycolate (450 mM). (**E**) Dose-dependent effect of thioglycolate addition over the production of IL18 in A431 cells, as quantified by ELISA. All the experiments in this figure were performed at least three times and analyzed in triplicates. Data are presented as mean (SEM, Mann-Whitney test). *P < 0.05, **P < 0.01.

SUPPLEMENTARY FIG 3

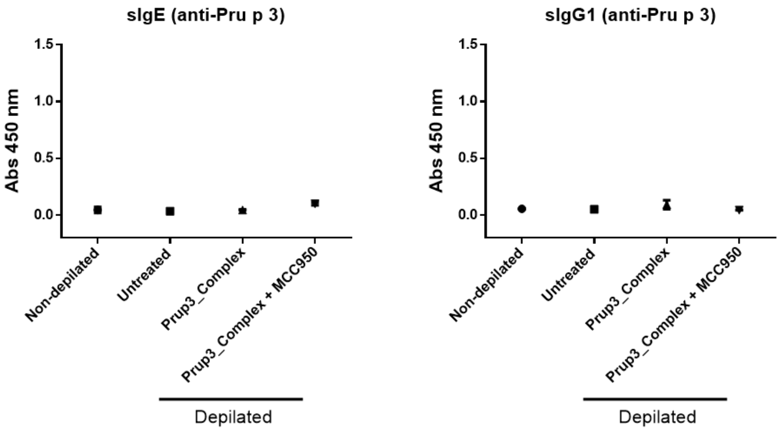

Supplementary Fig. 3. Prup3_complex exposure after depilation does not induce detectable allergen specific production in BALB/c mice. ELISA analysis showing no changes in sIgE and sIgG1 detection due to allergen exposure after depilation (n = 5 mice/ group). Each mice was analyzed in triplicates and mouse involved in these analyses come from two different independent sensitization protocols. Data are presented as mean (SEM, Mann-Whitney test).

SUPPLEMENTARY FIG 4

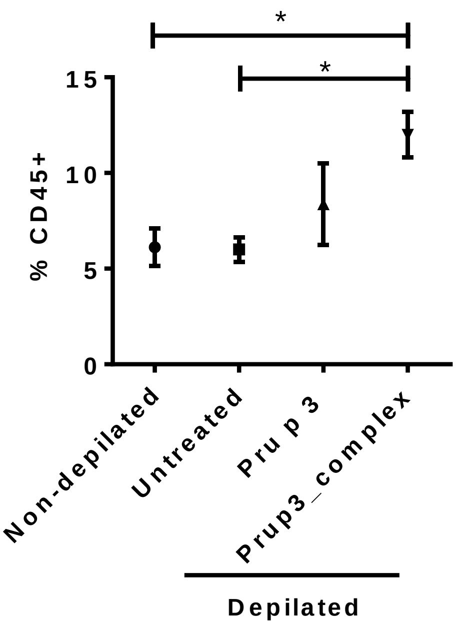

Supplementary Fig. 4. Exposure to Prup3_complex, but not depilation, induces recruitment of CD45+ cells to murine skins. Quantification of murine skins hybridized with anti-CD45 antibody. Nuclei were stained with DAPI. Quantification of CD45+ cell infiltration was calculated as the ratio between CD45+ and DAPI+ cells per image, using a Zeiss LSM 880 confocal microscope (n=5/ group; at least 3 sections were separately stained from each mouse at distal depths of the tissue and 3-5 images were taken per section). Data are presented as mean (SEM. Mann-Whitney test). *P < 0.05.

SUPPLEMENTARY FIG 5

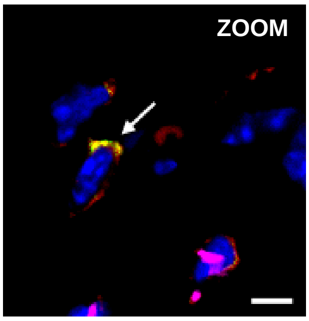

Supplementary Fig. 5. Zoom of the ILC-like cells presented in Fig. 5E, F. Experimental conditions already detailed in Fig. 5. Red: CD45, green: CD127, magenta: CD3, blue: DAPI. Bar = 5 µm.

SUPPLEMENTARY FIG 6

(A) (B) (C)

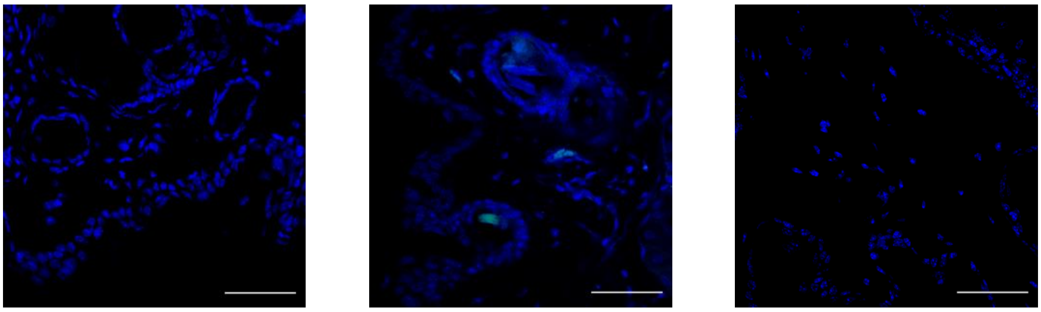

Supplementary Fig. 6. Isotype controls of the IF assays. Isotype controls for (A) CD207+, (B) CD3+ and (C) CD45+ CD127+ CD3- analyses. Experimental conditions already detailed in Fig. 5. Bar = 50 µm.

SUPPLEMENTARY FIG 7

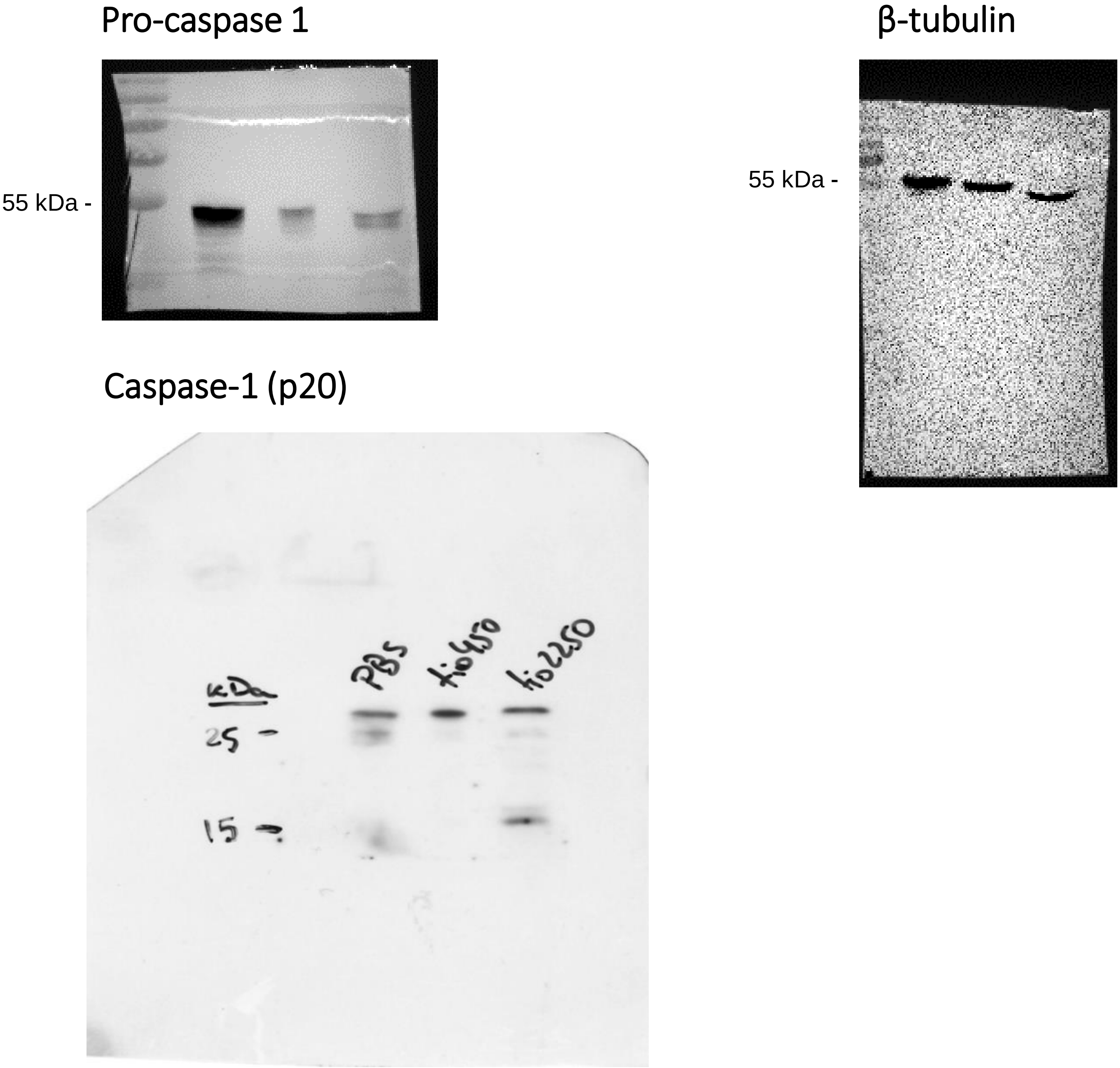

Supplementary Fig. 7. Uncropped images of Western-blot analyses showed in Fig. 2. Experimental conditions already detailed in Fig. 2.

SUPPLEMENTARY TABLE 1. List of antibodies used for the histological analyses.

Target	Concentration	Reference
**CD3**	1:100	17A2 (Invitrogen)
**CD45**	1:100	AF11 (R&D Systems)
**CD127**	1:50	A7R34 (Invitrogen)
**CD207**	1:100	eBioL31 (Invitrogen)
**NLRP3**	1:200	PA5-79740 (Invitrogen)

SUPPLEMENTARY TABLE 2. List of oligonucleotides used for the RTqPCR.

Gene	Oligonucleotide sequence (5'-3')	
***Ppia* Mouse**	**Forward**	TCAACCCCACCGTGTTCTTC
	**Reverse**	CCAGTGCTCAGAGCTCGAAA
***Nlrp3* Mouse**	**Forward**	GCGTGTTGTCAGGATCTCGCATTGG
	**Reverse**	GTGTCTCCAAGGGCATTGCTTCGTAG
***Il1b* Mouse**	**Forward**	TGGACCTTCCAGGATGAGGACA
	**Reverse**	GTTCATCTCGGAGCCTGTAGTG
***Il18* Mouse**	**Forward**	ACTGTACAACCGCAGTAATACGG
	**Reverse**	GCCAGTCCTCTTACTTCACTGTCTT
***Il25* Mouse**	**Forward**	GCAATGATCGTGGGAACCCACACCG
	**Reverse**	CAGGCATCGAGCGTGGTACAGGTC
***Il33* Mouse**	**Forward**	CTACTGCATGAGACTCCGTTCTG
	**Reverse**	AGAATCCCGTGGATAGGCAGAG
***Gapdh* Human**	**Forward**	GAGTCAACGGATTTGGTCGT
	**Reverse**	TTGATTTTGGAGGGATCTCG
***Il1b* Human**	**Forward**	AAACAGATGAAGTGCTCCTT
	**Reverse**	TGGAGAACACCACTTGTTGC
***Il18* Human**	**Forward**	GCTTGAATCTAAATTATCAGTCA
	**Reverse**	TGAAGATTCAAATTGCATCTTAT
